# Adenoid cystic carcinoma of the head and neck – treatment strategies of a highly malignant tumor with variable localizations

**DOI:** 10.18632/oncoscience.581

**Published:** 2023-06-28

**Authors:** Florian Dudde, Kai-Olaf Henkel, Filip Barbarewicz

**Keywords:** adenoid cystic carcinoma, treatment strategies, atypical localization

Head and neck tumors are among the most common malignancies [1]. In this anatomical region, squamous cell carcinoma (SCC) is the most common malignant entity [1]. However, there are also other malignant tumors that, unlike SCC, originate in the salivary glands of the head and neck region, such as mucoepidermoid carcinoma or adenoid cystic carcinoma (ACC) [2].

In general, tumors originating from the minor salivary glands often show a higher degree of malignancy than tumors of the major salivary glands (parotid gland, submandibular gland, sublingual gland) [2]. Consequently, the ACC in particular is often localized in the area of the hard palate (small salivary glands). In rare cases, ACC has also been described in other regions of the head and neck region, such as the paranasal sinuses or the tongue [3, 4].

The clinical features of ACC in the head and neck region are often variable. Typically, a swelling of the respective facial region that progresses quickly or slowly, depending on the growth pattern, is often associated with diffuse pain [5]. Furthermore, signs of paralysis and reduced sensitivity in the facial region are possible due to the perineural and perivascular pattern of ACC spread [5].

Most ACC usually show a slow growth pattern with a histologically highly differentiated cell picture, but markedly infiltrative growth behavior [5]. Histologically, the ACC often does not show any increased mitotic rates, which leads in particular to a lack of sensitivity towards chemotherapeutic agents [6].

In advanced ACC, even conventional radiation therapy can sometimes only achieve limited improvement with regard to the long-term outcome of ACC given the low mitotic aspect [6]. Diagnostically, three-dimensional imaging such as computed tomography and/or magnetic resonance imaging offer advantages with regard to tumor spread and the presence of metastases in the sense of tumor staging and provide important information in the context of therapy planning.

**Figure 1:**
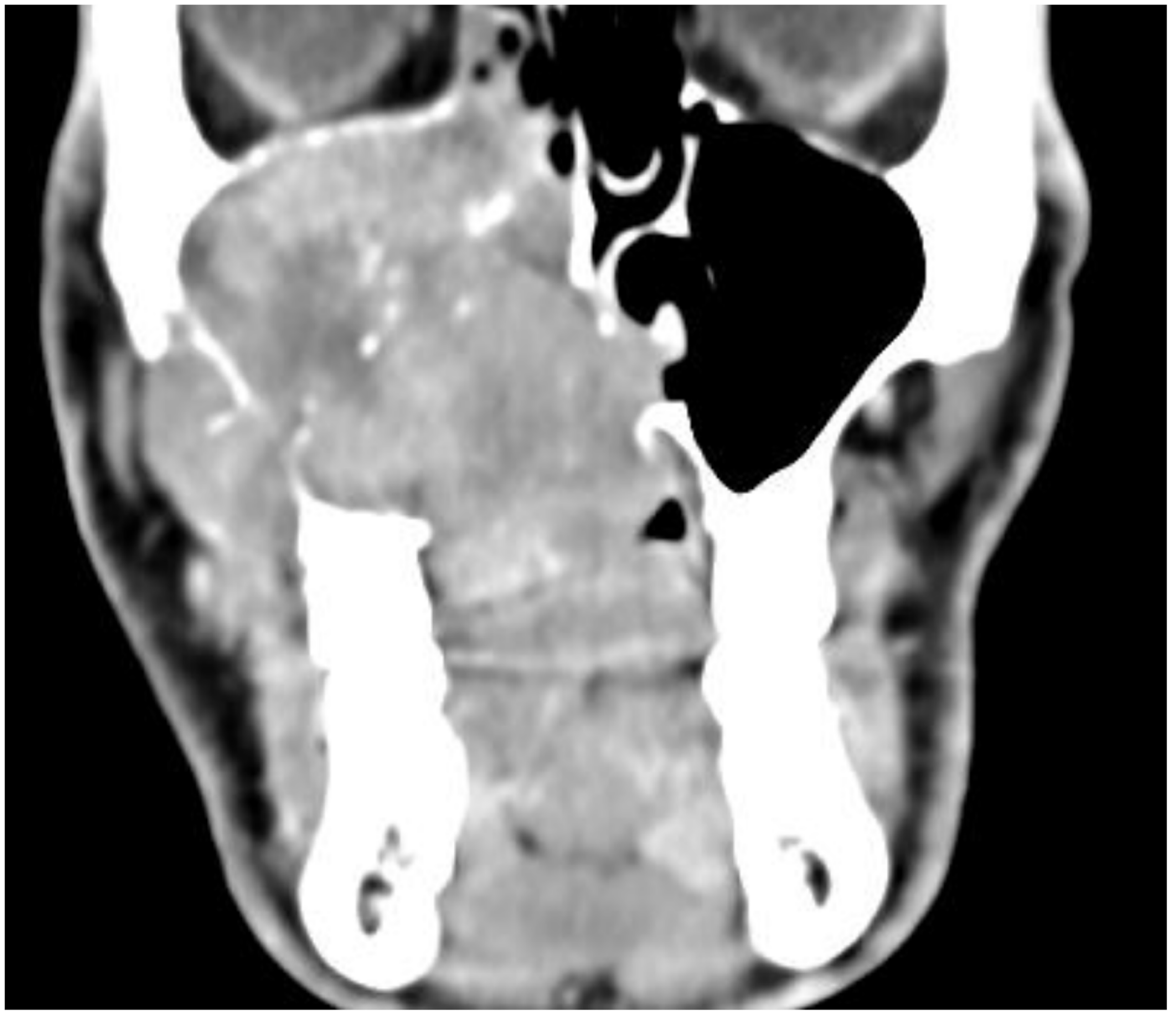
CT-scan in a coronal section showing an adenoid cystic carcinoma as an inhomogeneous mass in the right maxillary sinus with infiltration of neighboring structures.

Typically, ACC can show increased uptake of contrast medium agent with an infiltrative spread pattern in the respective anatomical region (Figure 1) [3]. However, it is difficult to clearly differentiate ACC from other malignancies of the head and neck region. The definitive diagnosis is usually made by confirming the histopathological findings in the context of an incisional biopsy.

Histopathologically, ACC can show a cribriform (most common), tubular, or solid growth pattern [5]. Furthermore, immunohistochemistry offers an essential advantage with regard to differentiation from other salivary gland malignancies [5].

The therapy of ACC in the head and neck region is sometimes challenging and depends in particular on the progression of the disease and the specific localization.

The treatment of choice, especially in localized tumor stages, consists of complete resection of the ACC with a sufficient safety margin (2–3 cm) and is usually supplemented by a neck dissection [7]. Due to the already noted perivascular and perineural growth pattern, an adequate safety margin may not be achieved in severe cases.

In addition, it must be noted that a sufficient safety margin sometimes cannot be achieved in the case of tumors in the head and neck region due to the anatomical conditions. Consequently, this leads to an increased risk of recurrence and/or residual disease in many malignancies of this region [7]. Furthermore, the reconstruction planned after the tumor resection in the respective head and neck region can be challenging. With regard to the plastic reconstruction of palatal defects in particular, various surgically challenging local and especially distant flaps (e.g., radialis flap) are sometimes suitable [8].

In addition, in sensitive locations with pronounced infiltration (e.g., maxillary sinus), adjuvant radiotherapy in the form of proton therapy can be helpful after surgical tumor resection [3, 9]. Young patients in particular can benefit from this new form of radiation therapy with regard to long-term survival if they have pronounced ACC findings [9].

A two-stage surgical procedure with initial tumor resection and epithesis treatment can sometimes be advantageous in therapy planning and plastic reconstruction with the above-mentioned distant flaps after adjuvant proton therapy [3]. However, since proton therapy is only available to a limited extent, close coordination between the treating departments is essential in order to provide the patient with a state-of-the-art therapy.

In general, the prognosis for ACC is acceptable with regard to 5-year survival of up to 80% [10]. However, addressed perivascular and perineural spreads as well as general lymphatic metastatic behavior significantly reduce survival rates [11].

It should be noted that head and neck ACC can occur in different anatomical regions. In principle, ACC grow slowly and consequently often cause a lack of clinical symptoms.

Their perivascular and perineural growth pattern can present therapeutic challenges, particularly in the head and neck region. The treatment of ACC in the head and neck region consists of surgical tumor resection with subsequent neck dissection and one- or two-stage plastic defect reconstruction. Severe ACC often require adjuvant radiotherapy. Proton therapy, as a special form of radiotherapy, seems to have decisive advantages with regard to the long-term survival of this highly malignant tumor originating from the small salivary glands.
